# Development a clinical prediction model of the neurological outcome for patients with coma and survived 24 hours after cardiopulmonary resuscitation

**DOI:** 10.1002/clc.23403

**Published:** 2020-06-23

**Authors:** Hai Wang, Long Tang, Li Zhang, Zheng‐Liang Zhang, Hong‐Hong Pei

**Affiliations:** ^1^ Emergency Department & EICU The Second Affiliated Hospital of Xi'an Jiaotong University Xi'an Shaan Xi China; ^2^ Department of Emergency Shaanxi Provincial People's Hospital Xi'an Shaan Xi China

**Keywords:** cardiac arrest, cardiopulmonary resuscitation (CPR), neurological function, prediction model

## Abstract

**Background:**

Cardiac arrest is still a global public health problem at present. The neurological outcome is the core indicator of the prognosis of cardiac arrest. However, there is no effective means or tools to predict the neurological outcome of patients with coma and survived 24 hours after successful cardiopulmonary resuscitation (CPR).

**Hypothesis:**

Therefore, we expect to construct a prediction model to predict the neurological outcome for patients with coma and survived 24 hours after successful CPR.

**Methods:**

A retrospective cohort study was used to construct a prediction model of the neurological function for patients with coma and survived 24 hours after successful CPR. From January 2007 to December 2015, a total of 262 patients met the inclusion and exclusion criteria.

**Results:**

The predictive model was developed using preselected variables by a systematic review of the literature. Finally, we get five sets of models (three sets of construction models and two sets of internal verification models) which with similar predictive value. The stepwise model, which including seven variables (age, noncardiac etiology, nonshockable rhythm, bystander CPR, total epinephrine dose, APTT, and SOFA score), was the simplest model, so we choose it as our final predictive model. The area under the ROC curve (AUC), specificity, and sensitivity of the stepwise model were respectively 0.82 (0.77, 0.87), 0.72and 0.82. The AUC, specificity, and sensitivity of the bootstrap stepwise (BS stepwise) model were respectively 0.82 (0.77, 0.87), 0.71, and 0.82.

**Conclusion:**

This new and validated predictive model may provide individualized estimates of neurological function for patients with coma and survived 24 hours after successful CPR using readily obtained clinical risk factors. External validation studies are required further to demonstrate the model's accuracy in diverse patient populations.

## 1 BACKGROUND

Cardiac arrest is still a global public health problem at present. The worldwide incidence rate of cardiac arrest is about 418/100 000. Cardiac arrest causes 15% to 20% of global deaths every year.^1^, ^2^ With the development and popularization of cardiopulmonary resuscitation (CPR), the restoration of spontaneous circulation (ROSC) has been dramatically improved, but the overall survival rate is still only about 10%. The neurological outcome is the core indicator of the prognosis of cardiac arrest.^3^ Among the patients with successful CPR, the rate of favorable neurological outcome was only 2.7% to 13.9%.^4^, ^5^, ^6^ Most of the patients with unfavorable neurological outcome eventually died or were in a vegetative state.^7^, ^8^ At present, related studies had shown that electroencephalogram, short‐delay somatosensory evoked potential (SSEEP), neuron‐specific enolase (NSE), S‐100B, microRNAs (miRNAs), near‐infrared spectroscopy (NIRS), and so forth may be used as a predictor of neurological function for patients with successful CPR.^9^, ^10^ The prediction of outcomes at 24 hours after ROSC may be clinically less important since patients after CPR. However, the 24‐hour survival of CPR is only about 10%, and their final prognosis is also uncertain. Only one third or fewer patients who survived 24 hours after successful CPR could discharge.^11^ At the same time, there is no effective means or tools to predict the neurological outcome of patients with coma and survived 24 hours after successful CPR. Patients with coma and survived 24 hours after successful CPR.

Therefore, we expect to construct a prediction tool that may be used to predict the neurological outcome for patients with coma and survived 24 hours after successful CPR.

## 2 METHODS

### 2.1 Study design

A retrospective cohort study.

### 2.2 Objective

To construct a prediction model that could predict the favorable neurological outcome of patients with coma and survived 24 hours after successful CPR**.**

### 2.3 Data source

The data in this study were provided by Fabio Silvio Taccone and stored in Dryad Database (https://datadryad.org/resource/doi:10.5061/dryad.qv6fp83).^12^, ^13^

### 2.4 The definition of successful CPR

(a) ECG monitoring showed effective cardiac rhythm, including sinus rhythm, borderline rhythm or accelerated ventricular autonomic rhythm; (b) palpable arterial pulsation; (c) under the condition of spontaneous breathing or mechanical ventilation, with or without drugs to maintain systolic pressure ≥60 mm Hg.

### 2.5 The definition of neurological outcome

(a) Favorable neurological outcome was considered as a cerebral performance category (CPC) 1‐2; (b) unfavorable neurological outcome as CPC 3‐5**.**

### 2.6 Inclusion criteria

(a) The patient was in a coma (Glasgow Coma Scale, GCS < 9); (b) Patients survived more than 24 hours after intensive care unit (ICU) admission.

### 2.7 Exclusion criteria

Patients with previous neurological impairment, including paralysis, muscle weakness, poor coordination, loss of sensation, seizures, confusion, pain, and altered levels of consciousness.

### 2.8 Participants

From January 2007 to December 2015, a total of 435 patients were with successfully CPR, 51 of whom died within 24 hours, 76 of whom without coma, and ultimately 308 of whom met the inclusion criteria. Forty‐six patients with previous neurological impairment were excluded. Finally, 262 patients met the inclusion and exclusion criteria.

### 2.9 Postresuscitation care

The protocol of postresuscitation management has been extensively described elsewhere, widely accepted, and applied. Briefly, all cardiac arrest patients with coma received targeted temperature management (TTM; target body temperature: 32‐34°C) for 24 hours. Rewarming (<0.5°C/h) was achieved passively. Midazolam, morphine, and cisatracurium were administered for deep sedation to control shivering. PiCCO technology was used to monitor hemodynamics. Repeated transesophageal and transthoracic echocardiography were used to assess cardiac function. Mean arterial pressure (MAP) was maintained at >65 to 70 mm Hg using volume resuscitation, dobutamine, or noradrenaline, whenever needed. Extracorporeal membrane oxygenation (ECMO) or intra‐aortic balloon counterpulsation (IABP) was also used in patients with severe cardiogenic shock. Mechanical ventilation was used to maintain SpO2 > 94% and normocapnia. Blood glucose was maintained at 110 to 150 mg/dL by a continuous insulin infusion. Enteral nutrition was initiated during TTM and continued after that according to gastric tolerance.

### 2.10 Clinical and biochemical data collection

The following data were collected on the day of admission: (a) demographics: age, sex, weight; (b) pre‐existing chronic diseases: chronic anticoagulation, chronic heart failure, hypertension, coronary artery disease, diabetes, chronic obstructive pulmonary disease (COPD), chronic renal failure and liver cirrhosis; (c) etiology of cardiac arrest and information regarding CPR: out of the hospital or hospital CPR, witnessed arrest, bystander CPR, initial rhythm, etiology of CPR, time to ROSC, total epinephrine dose, corticoids; (d) laboratory test index: LDH, APTT, Glu, PH, PO2, PCO2, MAP, Lac, CRP, creatinine and ScvO2/SvO2 (central venous oxygen saturation or mixed venous oxygen saturation); (e) severity of disease: SOFA (Sequential Organ Failure Assessment) and APACHE II score (Acute Physiology and Chronic Health Evaluation II score).

Special treatment including TTM, mechanical ventilation, continuous renal replacement therapy (CRRT), intra‐aortic balloon counterpulsation (IABP), and ECMO was collected during the ICU. (f) The CPC scale at 3 months was performed prospectively during follow‐up visits or by telephone interview with the general practitioner.

### 2.11 Selection of predictor variables

A review of literature was performed for the risk factors related to neurological outcomes in patients after CPR. The risk factors considered in the model are those that are deemed to be significantly related to neurological outcomes in patients after CPR and are readily available in clinical practice. Other risk factors may be worth including, but because they can only be measured by time‐consuming, expensive, or invasive testing procedures, these risk factors are usually not taken into account. To make these models as easy to use in clinical practice and minimize noise, we restrict our attention to those risk factors that are generally accepted, readily available, and precisely measured in clinical practice.

### 2.12 Statistical methods

(a) Statistical description: mean ± SD (*x* ± *s*). (b) The selected variables were used to construct the predictive model including multiple fractional polynomial models (MFP model: a method that it allows software to determine whether an explanatory variable was important for the model, and its functional form), full model, stepwise selected model (stepwise model: a method of fitting regression models in which the choice of predictive variables is carried out by an automatic procedure). (c) As a result that a total of 114 patients finally had a good neurological function, only 11 variables were chosen to construct and verify the models to avoid the model overfitting. (d) As the relatively small sample size of our study, we adopted bootstrapping for internal validation (bootstrap resampling 500 times) to verify the models. (e) The missing values in this database are ridiculously small (1‐2%), so there is no special handling of the missing values during model building. Statistical analysis was performed using Empower Stats version 2019 epidemiology software (www.empowerstats.com) and R software.

## 3 RESULTS

### 3.1 The clinical characteristics of patients

A total of 262 patients met the inclusion and exclusion criteria and were included in the study. The average age was 61.66 ± 15.82 years, male/female ratio was 70/192. Previous medical history included previous chronic anticoagulant (n = 50), chronic heart failure (n = 60), hypertension (n = 109), coronary heart disease (n = 113), diabetes (n = 64), COPD (n = 45), chronic renal failure (n = 41), cirrhosis (n = 12), nonshock arrhythmia (n = 137), and shock (n = 135). Among them, noncardiac arrest etiology (n = 86), out‐of‐hospital cardiac arrest (n = 155), witnessed cardiac arrest (n = 223), and bystander CPR (n = 168). The total epinephrine dose was 4.34 ± 3.90 mg; the time to ROSC was 19.22 ± 14.86 minutes. Finally, 114 patients had good neurological function recovery (Table 1).

**TABLE 1:** The clinical characteristics of patients

Variables	Mean ± SD/N (%)
Age, years	61.66 ± 15.82
Sex (F/M)	70/192
Chronic anticoagulation, n (%)	50 (19.08%)
Chronic heart failure, n (%)	60 (22.90%)
Hypertension, n (%)	109 (41.60%)
Coronary artery disease, n (%)	113 (43.13%)
Diabetes, n (%)	64 (24.43%)
COPD, n (%)	45 (17.18%)
Chronic renal failure, n (%)	41 (15.65%)
Liver cirrhosis, n (%)	12 (4.58%)
Nonshockable rhythm, n (%)	137 (52.29%)
Noncardiac etiology, n (%)	86 (32.82%)
Out of hospital, n (%)	155 (59.16%)
Witnessed arrest, n (%)	223 (85.11%)
Bystander CPR, n (%)	168 (64.12%)
Epinephrine total dose, mg	4.34 ± 3.90
Time to ROSC, min	19.22 ± 14.86
APTT, s	43.57 ± 29.92
LDH, IU/L	387.65 ± 226.63
INR	1.59 ± 1.29
Glucose, mg/dL	244.81 ± 117.81
pH	7.29 ± 0.13
PO2, mm Hg	156.57 ± 107.93
PCO2, mm Hg	38.70 ± 9.03
MAP, mm Hg	91.24 ± 21.40
Lac, mmol/L	6.17 ± 3.16
CRP, mg/L	56.71 ± 69.61
Creatinine, mg/dL	1.50 ± 1.25
Corticoids, n (%)	55 (20.99%)
IABP, n (%)	19 (7.25%)
ECMO, n (%)	31 (11.83%)
Mechanical ventilation, n (%)	261 (99.62%)
CRRT, n (%)	31 (11.83%)
SOFA score	10.68 ± 3.51
Length of ICU stay, days	7.95 ± 10.14
Favorable neurological outcome at 3 months, n (%)	114 (43.51%)

### 3.2 The results of univariate analysis and multivariate logistic regression analysis

A total of 11 variables selected by a systematic review of the literature are significantly related to neurological outcomes in patients after CPR and easily available in clinical practice. These variables including age, out of the hospital, bystander CPR, time to ROSC, total epinephrine dose, noncardiac etiology, nonshockable rhythm, APTT, mechanical ventilation, ScvO2/SvO2, and SOFA score were included in univariate analysis and multivariate logistic regression. Multivariate logistic regression analysis showed that the statistical effect of age, bystander CPR, epinephrine total dose, noncardiac etiology, nonshockable rhythm, APTT, ScvO2/SvO2, and SOFA score was statistically significant (Table 2).

**TABLE 2:** The results of the univariate and multivariate logistic regression analysis

Exposure	Univariate OR (95% CI), *P*	Multivariate OR (95% CI), *P*
Age (year)	0.979 (0.966, 0.993), .004	0.971 (0.953, 0.988), .001
Out of hospital		
N0	Reference	Reference
Yes	1.010 (0.658, 1.551), .962	1.024 (0.548, 1.915), .940
Bystander CPR		
N0	Reference	Reference
Yes	2.046 (1.269, 3.298), .003	2.183 (1.141, 4.177), .018
Time to ROSC (min)	0.976 (0.959, 0.992), .004	0.990 (0.958, 1.023), .552
Epinephrine total dose (mg)	0.866 (0.806, 0.930), <.001	0.816 (0.708, 0.940), .005
Noncardiac etiology		
N0	Reference	Reference
Yes	0.551 (0.354, 0.860), .009	0.513 (0.282, 0.934), .029
Nonshockable rhythm		
N0	Reference	Reference
Yes	0.289 (0.184, 0.452), <.001	0.295 (0.165, 0.531),<.001
APTT (s)	1.004 (0.996, 1.012), .316	1.017 (1.006, 1.028), .002
Mechanical ventilation		
N0	Reference	Reference
Yes	0.000 (0.000, Inf), .980	0.000 (0.000, Inf), .986
ScvO2/SvO2	0.970 (0.946, 0.994), .015	0.969 (0.940, 0.999), .042
SOFA score	0.851 (0.796, 0.910), <.001	0.812 (0.749, 0.881), <.001

### 3.3 Prediction model construction

**FIGURE 1:**
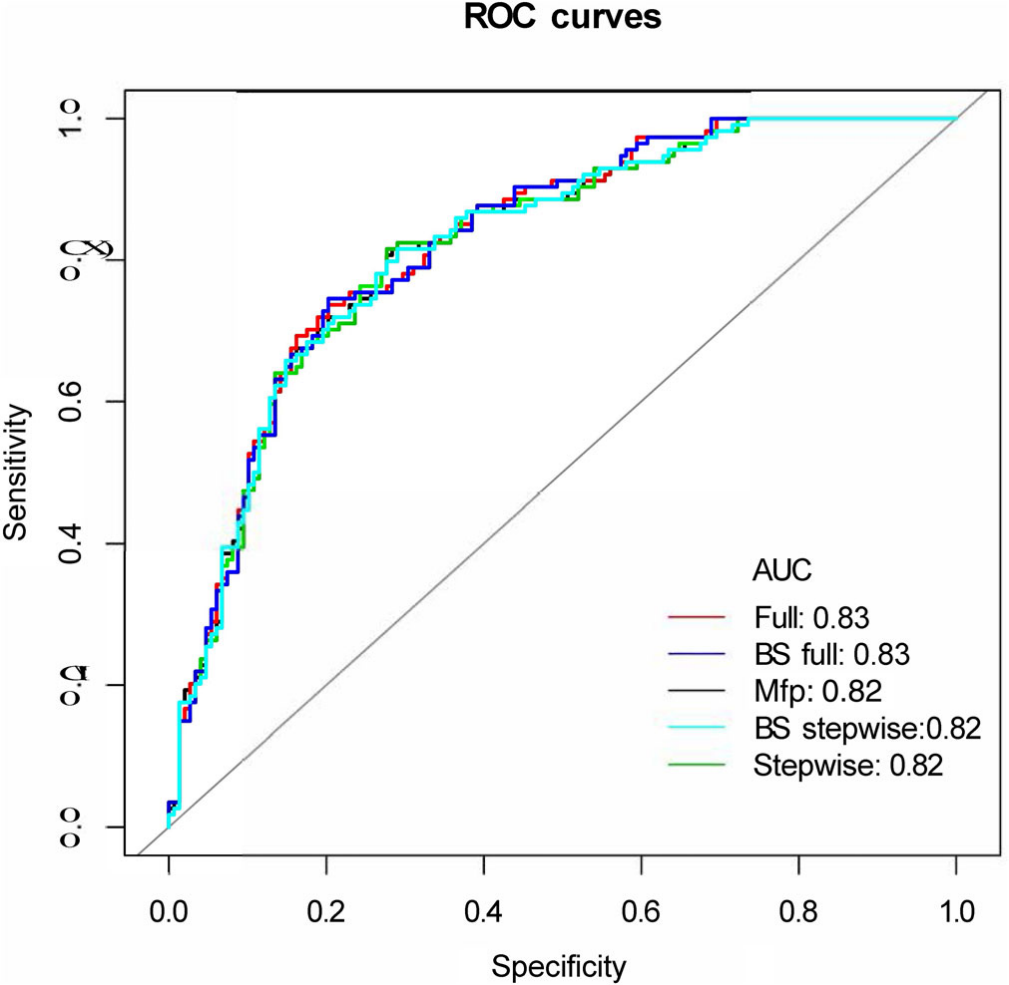
The ROC curve of the predictive model

The predictive model was developed using the 11 variables selected by a systematic review of the literature. We finally constructed a total of three prediction models, including the MFP model (multiple fractional polynomial model), full model, and stepwise model. The AUC of MFP model, full model and stepwise model were respectively 0.82 (0.77, 0.87), 0.83 (0.77, 0.88), and 0.82 (0.77, 0.87) (Table 3 and Figure 1).

**TABLE 3:** The results of predictive models

	MFP model	Full model	Stepwise model	Bootstrap full	Bootstrap stepwise
AUC	0.82 (0.77, 0.87)	0.83 (0.77, 0.88)	0.82 (0.77, 0.87)	0.82 (0.77, 0.88)	0.82 (0.77, 0.87)
Specificity	0.72	0.80	0.72	0.80	0.71
Sensitivity	0.82	0.74	0.82	0.75	0.82
Accuracy	0.76	0.77	0.76	0.77	0.76

*Note:* MFP model (multiple fractional polynomial model); stepwise selected model (stepwise model); bootstrap full (full model from bootstrap); bootstrap stepwise (BS stepwise, stepwise most selected model from bootstrap).

### 3.4 Verification of prediction model

**FIGURE 2:**
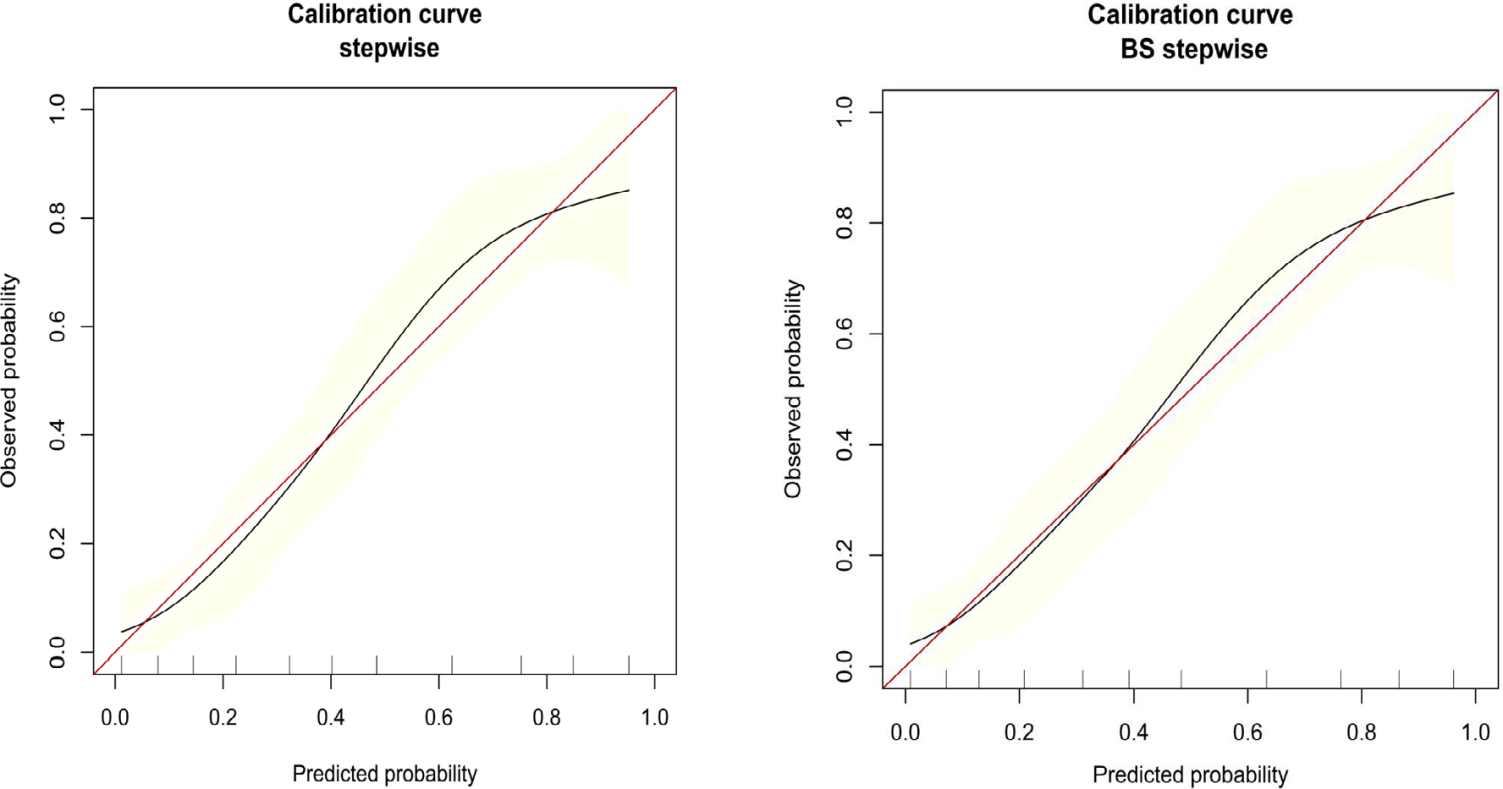
The calibration curve of the stepwise model and bootstrap (BS) stepwise model

As the relatively same sample size of our study, we adopted bootstrapping for internal validation. The bootstrap full model and bootstrap stepwise model were, respectively, used to verify further the accuracy and value of the full model and stepwise model. The AUC of bootstrap full (BS full) model and bootstrap stepwise (BS stepwise) model were respectively 0.82 (0.77, 0.88) and 0.82 (0.77, 0.87). The accuracy of the two validation model and the three predictive models were generally consistent. The stepwise model, which including seven variables (age, noncardiac etiology, nonshockable rhythm, bystander CPR, total epinephrine dose, APTT, and SOFA score), was the simplest model. The calibration curve of the stepwise model and bootstrap stepwise model also showed that the predicted probability and the observed probability were generally fitting. So we finally choose stepwise model as our target predictive model and construct a nomogram based on stepwise model (Table 3, Figures 1, 2, and S1).

## 4 DISCUSSION

We used the 11 variables selected by a systematic review of the literature to constructed three predictive models and adopted bootstrapping for internal validation. Finally, we got a simplest and higher accurate prediction model, including seven variables (age, noncardiac etiology, nonshockable rhythm, bystander CPR, total epinephrine dose, APTT, and SOFA score). Finally, we constructed a nomogram based on this model that may be used to predict the neurological function for patients with coma and survived 24 hours after successful CPR.

All the variables included in our final choice of stepwise model, related studies, have shown that they were firmly associated with the neurological outcome of patients after CPR and were very easy to obtain indicators in a clinical symbol.

Age is not only a significant risk factor for cardiac arrest but also an important prognostic factor for patients with cardiac arrest. As one grows older, the risk suffering from diabetes,^14^ hypertension,^15^ cardiovascular, and cerebrovascular diseases increased.^16^ These diseases are also significant risks of cardiac arrest.^17^

Cardiac etiology was the most common reason for cardiac arrest, about 50% to 60% of which is induced by heart‐related diseases, such as myocardial infarction, arrhythmia, or cardiac failure, and so forth.^18^ A retrospective study included 1041 patients with cardiac arrest, 63% of which were with cardiac etiology, and only 37% were with noncardiac etiology. This study found that the prognosis of patients with cardiac etiology was significantly better than the patients with noncardiac etiology (44% vs 23%, *P* < .01).^19^ The possible reasons were: (a) Patients with cardiac etiology were often complicated with heart‐related diseases in the past. A more accurate diagnosis could be obtained through a brief medical history, which could be provided by patients themselves, their family members, or their private doctors. Then the targeted treatment could be given quickly and accurately. (b) Noncardiac etiology arrest, the most common was respiratory related cardiac arrest. These patients needed relatively more time from the occurrence of anoxia symptoms to the initiation of cardiac arrest. When the patient had a cardiac arrest, the function of the lung was already inferior. When these patients suffered from cardiac arrest, although the pump function of the heart could be maintained through continuous and effective chest compressions; however, even if tracheal intubation and ventilator were given in time, hypoxemia could not be quickly relieved, and more importantly, a simple respirator was the first and most frequently received respiratory support devices, which with poor respiratory support.^20^

Although relevant studies had shown that initial shockable rhythm was closely associated with the prognosis of patients with cardiac arrest,^21^, ^22^, ^23^ We used a nonshockable initial rhythm instead of initial shockable rhythm in our study. The reasons were as follows: (a) nonshockable initial rhythm could be quickly recorded before cardiac arrest occurred because nonshockable initial rhythm taken a relatively long time to develop into cardiac arrest^24^; (b) the most of the initial shockable rhythm of cardiac arrest were developed from nonshockable initial rhythm^24^; (c) The time that initial shockable rhythm developed into cardiac arrest was concise, as a result, that the discover and diagnose the initial shockable rhythm with very difficult neither in or out of the hospital. Finally, the initial shockable rhythm could not be recorded adequately; (d) Related studies also showed that nonshockable initial rhythm was an independent risk factor for the prognosis of patients with cardiac arrest.^24^, ^25^

Bystander CPR had been widely proved to be related to the prognosis of patients with CPR. Bystander CPR could improve the ROSC and the forecast of the neurological function of patients after CPR.^26^, ^27^

Epinephrine was an essential drug for the treatment of cardiac arrest, and its dosage was strictly related to the prognosis of patients with cardiac arrest.^28^, ^29^ In the process of CPR, the more epinephrine, the less response to epinephrine and the more side effects of epinephrine.^30^, ^31^

Due to blood stop flowing and endothelial cell hypoxia damage after cardiac arrest, coagulation pathways would be activated, including endogenous and exogenous coagulation pathways, resulting in thrombosis. Thrombosis would further aggravate cerebral ischemia and hypoxia. Therefore, the coagulation status in the body after cardiac arrest was closely related to the neurological function of patients after CPR. PT or INR was an indicator of the exogenous coagulation pathway, while APTT was an indicator of the endogenous coagulation pathway.^32^ Our study found that APTT was firmly related to the neurological prognosis of cardiac arrest. The reason was that the function of the endogenous coagulation pathway was mainly reflected inactivating the coagulation system, while the waterfall reaction of the coagulation system was manifested primarily on the endogenous coagulation pathway. Besides, platelets were also an essential part of thrombosis.

SOFA score was currently used for organ failure score and was widely used for the prognosis prediction of critically ill patients.^33^, ^34^, ^35^ A study including 173 out‐of‐hospital CPR patients treated with hypothermia found that the higher the SOFA score, the higher the mortality rate and the worse neurological function during hospitalization.^36^

MFP can be used to investigate whether the relationship between covariables and independent variables is nonlinear.^37^ In our research, we have constructed three prediction models, including the MFP model, full model, and the stepwise model. The accuracy of the MFP model and the remaining two models were consistent, indicating that there was no nonlinear relationship between the covariables and independent variables included in the model, so the results of the full model and stepwise model were reliable. The variables in our model were selected according to the literature review, which avoids that the *P*‐value was not significant due to the small sample size, which led to the crucial variables that were not included in the prediction model. At the same time, we found that the SOFA score included the mean arterial pressure (MAP), and the content of the SOFA score and APACHE II score was partially repeated, and the most important was that the SOFA score was simpler than APACHE II. To avoid over‐fitting of the model and obtain a simpler model, we only included the SOFA score. Therefore, we did not include the MAP and APACHE II scores for the construction and verification of the model. Also, there were a total of 114 patients had a good neurological function, and therefore only 11 variables were selected to construct and verify the models to avoid the model overfitting. The results of the calibration curve also showed that the stepwise model and bootstrap stepwise model was reliable. To sum up, we construct a relatively simple, reliable, and good clinical practice prediction model.

Compared with Seewald S′ models, it seemed that the accuracy of our prediction model was relatively lower. However, the study population of Seewald S′ model was out of hospital cardiac arrest, and our study population was patients with coma and 24 hours after CPR. Therefore, our study still had value in predicting the prognosis in the specific population.^38^

### 4.1 The application of this study

(a) Although the sample size of our study was relatively small, the rate of ROSC was deficient, and very fewer patients could survive for 24 hours. Therefore, it was challenging to collect 262 coma patients with cardiac arrest, ROSC, and 24‐hour survival. (b) At the same time, a nomogram had been constructed in our study, which could be used to predict the prognosis of neurological function for each individual who survived more than 24 hours. (c) How to use the nomogram. Each variable in the figure was marked with a scale on the line segment, representing the value range of them, and the length of the line segment reflected their contribution to the outcome event. The point in the figure, represented the single score corresponding to them under different values. Add their single score to get the total point. Finally, we could get the rate of favorable neurological outcome at 3 months according to the total point. Through the above methods, we had constructed a relatively stable, clinically essential, and straightforward prediction model.

### 4.2 Limitations of research

(a) Retrospective data were used in the model construction and verification in this study, so there was a specific potential bias risk, which needed further prospective experiments to verify this prediction model. (b) Since the construction and verification of the model were from a single‐center, the prediction value would decline outside this hospital, so this prediction model would need more studies to confirm it.

## 5 CONCLUSION

This new and validated predictive model may provide individualized estimates of neurological function for patients with coma and survived 24 hours after successful CPR using readily obtained clinical risk factors. External validation studies are required further to demonstrate the model's accuracy in diverse patient populations.

## CONFLICT OF INTEREST

The authors declare no potential conflict of interests.

## ETHICS STATEMENT

New ethics approval was not applicable, because the original author had obtained the ethical approval when conducting this study. Permission to participate was also not appropriate, because our review was a retrospective study of data reuse, and the message of the patients was anonymous.

## Supporting information


**Figure S1** The nomogram of the stepwise model.

## Data Availability

The data that support the findings of this study are openly available in [Dryad] at https://datadryad.org/resource/doi:10.5061/dryad.qv6fp83, Reference 13.
