# Supplementary figures and images for: Development a clinical prediction model of the neurological outcome for patients with coma and survived 24 hours after cardiopulmonary resuscitation
Source: Clin Cardiol. 2020 Jun 23;43(9):1024–31. doi: 10.1002/clc.23403 (PMC7462189; doi:10.1002/clc.23403)

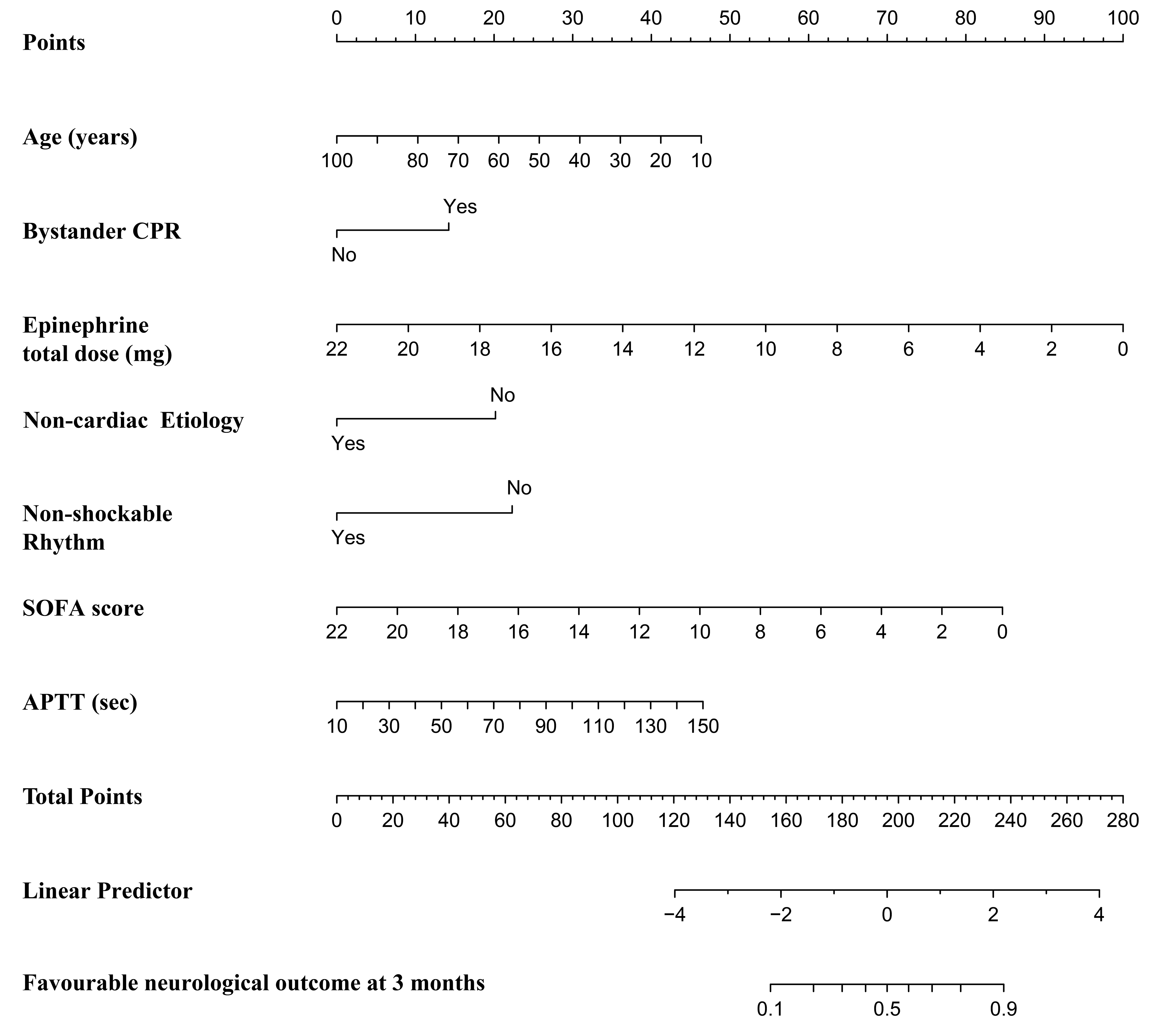

Supplement: Supplementary file 1 — Figure S1 The nomogram of the stepwise model. [file CLC-43-1024-s001.tif]
